# New and Delayed Artifactual Hypoglycemia Following Septic Shock in Two Scleroderma Patients

**DOI:** 10.7759/cureus.41900

**Published:** 2023-07-14

**Authors:** Alex Guzner, Emil Heinze, Lakshmi Sadasivam

**Affiliations:** 1 Internal Medicine, University of California Los Angeles David Geffen School of Medicine, Los Angeles, USA; 2 Endocrinology, Olive View University of California Los Angeles Medical Center, Los Angeles, USA; 3 Hospital Medicine, Olive View University of California Los Angeles Medical Center, Los Angeles, USA

**Keywords:** factitious hypoglycemia, fingerstick, patient health safety, capillary blood glucose, systemic scleroderma

## Abstract

The term artifactual hypoglycemia refers to a discrepancy between plasma glucose levels and what is noted on fingerstick glucose checks. In this report, we discuss the cases of two patients with scleroderma and Raynaud’s phenomenon who developed artifactual hypoglycemia while recovering from critical illness. In both cases, validation by earlobe measurements helped avoid further escalation of care and potential patient safety issues. There have been previous reports of artifactual hypoglycemia in this patient population, but the unique timing of symptom onset in these two patients provides a more nuanced understanding of the pathophysiology of this phenomenon.

## Introduction

Artifactual hypoglycemia, described as a discrepancy between laboratory values and true blood glucose levels [1], has been rarely documented in the existing literature. As per Whipple's criteria, symptoms or signs of hypoglycemia, plasma glucose levels less than 55 mg/dL, and resolution of symptoms with the restoration of normal glucose levels are necessary for the diagnosis of true hypoglycemia. However, low fingerstick glucose values in individuals sometimes trigger a call for a rapid response team, an extensive workup for hypoglycemia, initiation of treatment for hypoglycemia, such as 10% intravenous dextrose infusion, 50% dextrose injections or intravenous glucagon, and may lead to anxiety/confusion on the part of the patient, nursing staff, and physicians.

Earlobe measurement is an easy way at the bedside to corroborate low fingerstick glucose values while awaiting plasma glucose levels for the confirmation of artifactual hypoglycemia [2]. The earlobe lacks cartilage tissue and is not hence affected by the microvascular changes affecting scleroderma; hence, it is often used for the evaluation of hypoxia in scleroderma patients when finger pulse oximetry is deemed unreliable. The exact mechanism behind artifactual hypoglycemia is unclear but it has been attributed to physiological changes to extremity microcirculation in systemic sclerosis leading to slow transit and increased extraction of glucose.

Cases of artificial hypoglycemia in scleroderma and Raynaud’s phenomenon have been reported in the literature [1,3,4], and thus far the most likely mechanism appears to be peripheral vasoconstriction leading to decreased availability of glucose in capillary beds at extremities [5]. In this report, we discuss the cases of two patients with scleroderma who were treated in two different academic institutions and exhibited new-onset artifactual hypoglycemia following recovery from critical illness. In reviewing the common threads and differences between these two patients, we hope to further elucidate how this phenomenon occurs, propose a new pathophysiologic mechanism, and shed light on a high-risk time when hospitalized patients may be more likely to develop artifactual hypoglycemia.

## Case presentation

Case 1

A 60-year-old female with a past medical history of mixed connective tissue disease (MCTD) comprising diffuse scleroderma, systemic lupus erythematosus, and myositis presented with respiratory difficulties and hypotension and was eventually diagnosed with undifferentiated shock. This was attributed to supraventricular tachycardia, sepsis, and possible pulmonary embolism. She was admitted to the ICU and was treated with a norepinephrine drip, amiodarone drip, furosemide, vancomycin, cefepime, and heparin drip. She also had acute kidney injury but did not require dialysis. Her home medications were prednisone, aspirin, atorvastatin, colchicine, cyclobenzaprine, albuterol via nebulizer, calcium carbonate, famotidine, folic acid, furosemide, magnesium oxide, mycophenolate, metoclopramide, pantoprazole, and ursodiol. Notably, she had Raynaud’s phenomenon involving fingertips and toes without digital ulcerations. She did not have a personal or family history of diabetes. The patient had poor oral intake and her blood glucose on basic metabolic panel testing ranged from 70 to 110 mg/dl. Hence, fingerstick glucose monitoring was initiated for early detection of hypoglycemia. On hospital day six, she was noted to have a fingerstick glucose of 49 mg/dl and was started on intravenous 10% dextrose for a few hours. The following day, she had fingerstick blood glucose levels close to 20 mg/dl despite using a different glucometer. However, she did not exhibit any hypoglycemic symptoms at any time. She received four doses of 50 ml 50% dextrose in water and 1 mg glucagon injection intravenously. Yet, all fingerstick glucose levels remained very low at below 20 mg/dl. Given that there was a complete absence of hypoglycemic symptoms, no further treatment was provided. In the meantime, a quick literature search revealed a few similar events documented in scleroderma patients. Hence, a simultaneous plasma glucose test was done, which showed a value of 371 mg/dl while the concomitant finger stick value was less than 10 mg/dl. In addition, checking glucose levels from the earlobe using the same glucometer yielded normal levels that were very close to plasma glucose values. A diagnosis of artifactual hypoglycemia was therefore established.

Case 2

An 89-year-old male with a history of systemic sclerosis complicated by interstitial lung disease on home oxygen, type I pulmonary hypertension (PAH), and severe aortic stenosis was admitted to the hospital with a diagnosis of septic shock and hypercarbic respiratory failure due to aspiration pneumonia and urosepsis. He was treated with oxygen via a high-flow nasal cannula, piperacillin-tazobactam, norepinephrine, and vasopressin. He was admitted to the ICU. His home medications were ambrisentan, tadalafil, treprostinil, prednisone, trimethoprim-sulfamethoxazole, furosemide, finasteride, and omeprazole. He had no personal or family history of diabetes. He was also started on stress-dose steroids due to additional concern for adrenal insufficiency due to the longstanding use of home prednisone. Morning cortisol levels the following day were within normal limits, but stress-dose steroids were continued given the severity of his illness. Over the next several days, his shock and respiratory status improved, and he was weaned off of pressors, high-flow oxygen treatment, and on hospital day five, his stress-dose steroids were stopped and he was put back on his home prednisone. On hospital day seven, he had a fingerstick glucose level of 37 mg/dl, without any evidence of hypoglycemic symptoms, which rose to above 150 mg/dl after treatment with a single dose of 50 ml 50% dextrose solution in water. The following day, fingerstick glucose level continued to be low at 60 mg/dl or less in all fingers, and despite the lack of any symptoms, he was started on a 10% intravenous dextrose infusion. While on the 10% intravenous dextrose infusion, his fingerstick glucose level continued to be lower than 60. A simultaneous comparison was made by checking glucose levels via glucometer at the earlobe and via venipuncture for plasma glucose levels. Both were demonstrated to be over 100 mg/dl. At this point, the 10% dextrose intravenous infusion was stopped, and glucose checks were only performed via earlobe checks. His subsequent values never fell below 90 mg/dl.

## Discussion

By reporting these two instances of artifactual hypoglycemia in scleroderma patients, we hope to raise awareness of this entity among clinicians, thereby preventing unnecessary treatments with potential patient safety implications. We also endeavor to analyze the potential underlying pathophysiology that might have led to this event occurring a few days after their critical illness, in fact during their recovery period. There are certain correlations between the two cases, the analysis of which may contribute to a more complex and nuanced understanding of this phenomenon.

Both patients presented with presumed septic shock, spent time in the ICU on vasopressors and antibiotics, and both developed artifactual hypoglycemia around the same point during their hospitalization after they had clinically improved. Both had completed or nearly completed a course of antibiotics and had been weaned off all vasopressors. Notably, prior to registering low fingerstick glucose levels, both had received multiple fingerstick checks per day, raising the possibility of endothelial trauma being a factor here.

Extremity trauma

Physiologic evidence has long demonstrated that extremity trauma can lead to vasoconstriction in patients with traumatic vasospastic disease or Raynaud’s [6]. The mechanism behind vasospasm may be due to a central sympathetic reflex [6], related to local vasospasm stemming from endothelial damage [7], or a combination of both central and peripheral effects [8].

Each time a patient receives a fingerstick glucose check, that patient experiences a small degree of endothelial trauma due to the puncture of the capillary to obtain blood. The pathogenesis of vasoconstriction due to systemic sclerosis is thought to be related to endothelial cell injury [9] followed by loss of mediators of vasodilation such as prostacyclin and nitric oxide [10]. Endothelial injury in all patients results in the release of the vasoconstrictor endothelin-1 (ET-1) [11], which acts locally on the vascular endothelium. In both patients with systemic sclerosis and Raynaud’s phenomenon, plasma ET-1 levels have been found to be increased [12]. 

Based on this information, it is not unreasonable to assume that multiple fingerstick tests leading to endothelial microtrauma in these vulnerable patients may have resulted in an exaggerated ET-1 response without compensation by vasodilators such as prostacyclin and nitric oxide, leading to local vasoconstriction and therefore artifactual hypoglycemia. The fact that these patients had low levels of fingerstick glucose in all fingers at the same time suggests that a component of this artifactual hypoglycemia is due to an upstream response owing to a sympathetic reflex, as described earlier, although this mechanism is not yet clearly elucidated.

Septic shock and systemic vasodilation 

The other common factor between these two patients was the presence of sepsis. Both patients presented with presumed septic shock requiring vasopressor support and developed artifactual hypoglycemia several days after treatment for the above; in fact, after they had improved hemodynamically. It is worth considering that perhaps the resolution of vasodilation due to sepsis may have contributed to a relative/rebound vasoconstriction of these patients’ extremities leading to the observed artifactual hypoglycemia. There is a possibility that their underlying vasoconstriction was masked by systemic vasodilation during active sepsis, but they had not exhibited artifactual hypoglycemia in the past as per reports from the patients or medical records pertaining to this. To more thoroughly evaluate this hypoglycemic phenomenon, it would be helpful to discuss the pathophysiology of sepsis. 

It is well understood that sepsis leads to systemic vasodilation due to profound inflammation [13] potentially in the setting of increased nitric oxide production by the enzyme inducible nitric oxide synthetase (iNOs) leading to persistent relaxation of vascular smooth muscle [13]. In addition to nitric oxide, many other pro-inflammatory cytokines are released, including the vasoconstrictor ET-1 [14]. Experimental models have demonstrated that nitric oxide and ET-1 are elevated at different times throughout the septic response, with the vasodilator nitric oxide contributing to the early phase of sepsis, while ET-1 dominates in late sepsis leading to organ dysfunction [15]. Research also indicates that in mice models with elevated nitric oxide levels, arterioles are hyporesponsive to the vasoconstriction of ET-1 until nitric oxide levels decrease [16]. In studies of plasma concentration of ET-1 in septic rats treated with antibiotics and fluids, these treatments reduced mortality but did not affect plasma levels of ET-1, suggesting that levels remain elevated even when sepsis is adequately treated [17]. When all of this logic is applied in the context of patients with systemic sclerosis with Raynaud’s phenomenon, it raises an interesting pathophysiologic phenomenon to explain the potential development of artifactual hypoglycemia.

As discussed earlier, patients with systemic sclerosis are known to have elevated levels of ET-1 [12] and loss of the vasodilators prostacyclin and nitric oxide [10,16]. When these patients become septic, it is likely that both nitric oxide and ET-1 are produced in excess, although there is no experimental evidence for how the levels of these two mediators compare in these patients compared to controls. In the natural course of sepsis, nitric oxide appears to predominate early and ET-1 later [15]. In patients who simultaneously receive fingerstick glucose checks, as they progress later into their sepsis course, they have both local [11] and systemic [14] drive for the production of ET-1. As nitric oxide production wanes and ET-1 dominates both locally and systemically, in patients known to have baseline elevated levels of ET-1 and decreased levels of nitric oxide, there is likely to be a very high ratio of vasoconstrictor to vasodilator production both locally in the fingertips and systemically. In this setting, it appears reasonable that late in the course of sepsis or as the vasodilatory component of sepsis begins to improve, peripheral vasoconstriction leading to decreased perfusion to the fingertips and artifactual hypoglycemia could be triggered to manifest.

## Conclusions

In this report, we have proposed two overlapping mechanisms behind the development of artifactual hypoglycemia. To conclude, in patients with systemic sclerosis with Raynaud’s phenomenon, any fingerstick glucose value less than 70 mg/dl with an absence of symptoms of hypoglycemia should be validated by an earlobe glucose check or venous check before administering intravenous dextrose, intravenous glucagon, etc., as well as embarking on an extensive workup for persistent hypoglycemia. In addition, clinicians must pay close attention to extremity perfusion when these patients develop sepsis due to alterations in the already delicate balance between vasoconstrictors and vasodilators. We also propose using earlobe sticks for glucose checks in this unique patient population in general.
